# (4-Bromo­phen­yl)(1-phenyl­sulfonyl-1*H*-indol-2-yl)methanone

**DOI:** 10.1107/S160053681004198X

**Published:** 2010-10-30

**Authors:** G. Chakkaravarthi, R. Panchatcharam, V. Dhayalan, A. K. Mohanakrishnan, V. Manivannan

**Affiliations:** a Department of Physics, CPCL Polytechnic College, Chennai 600 068, India; b Department of Research and Development, PRIST University, Vallam, Thanjavur 613 403, Tamil Nadu, India; c Department of Organic Chemistry, University of Madras, Guindy Campus, Chennai 600 025, India

## Abstract

In the title compound, C_21_H_14_BrNO_3_S, the indole ring system forms dihedral angles of 65.64 (8) and 59.30 (8)°, respectively, with the phenyl and bromo­phenyl rings. In the crystal, mol­ecules are connected by a C—H⋯O hydrogen bond, forming a chain along [101]. The chains are further connected by weak inter­molecular C—H⋯π inter­actions, forming a layer parallel to the *ac* plane.

## Related literature

For the biological activity of indole derivatives, see: Joshi & Chand (1982 ▶); Pomarnacka & Kozlarska-Kedra (2003 ▶); Poter *et al.* (1977 ▶). For related structures, see: Chakkaravarthi *et al.* (2007 ▶, 2008 ▶). For details of the configuration at the S atom, see: Bassindale (1984 ▶). For details of N-atom hybridization, see: Beddoes *et al.* (1986 ▶).
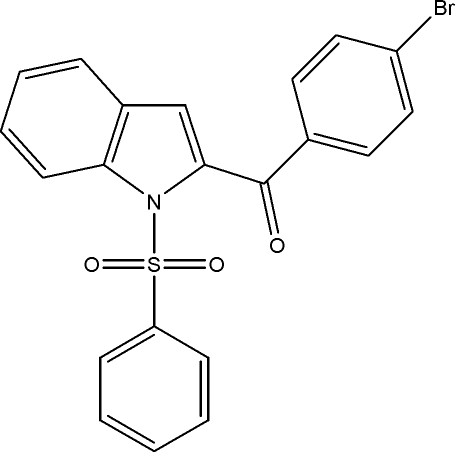


## Experimental

### 

#### Crystal data

C_21_H_14_BrNO_3_S
*M* *_r_* = 440.30
Monoclinic, 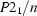

*a* = 8.482 (3) Å
*b* = 25.780 (4) Å
*c* = 8.690 (3) Å
β = 93.388 (3)°
*V* = 1896.9 (10) Å^3^
*Z* = 4
Mo *K*α radiation
μ = 2.30 mm^−1^
*T* = 295 K
0.24 × 0.22 × 0.20 mm

#### Data collection

Bruker Kappa APEXII diffractometer
Absorption correction: multi-scan (*SADABS*; Sheldrick, 1996 ▶) *T*_min_ = 0.609, *T*_max_ = 0.656
18123 measured reflections
4736 independent reflections
2944 reflections with *I* > 2σ(*I*)
*R*_int_ = 0.035

#### Refinement

*R*[*F* ^2^ > 2σ(*F* ^2^)] = 0.044
*wR*(*F* ^2^) = 0.111
*S* = 1.03
4736 reflections
244 parameters
H-atom parameters constrained
Δρ_max_ = 0.62 e Å^−3^
Δρ_min_ = −0.46 e Å^−3^

### 

Data collection: *APEX2* (Bruker, 2004 ▶); cell refinement: *SAINT* (Bruker, 2004 ▶); data reduction: *SAINT*; program(s) used to solve structure: *SHELXS97* (Sheldrick, 2008 ▶); program(s) used to refine structure: *SHELXL97* (Sheldrick, 2008 ▶); molecular graphics: *PLATON* (Spek, 2009 ▶); software used to prepare material for publication: *SHELXL97*.

## Supplementary Material

Crystal structure: contains datablocks I, global. DOI: 10.1107/S160053681004198X/is2617sup1.cif

Structure factors: contains datablocks I. DOI: 10.1107/S160053681004198X/is2617Isup2.hkl

Additional supplementary materials:  crystallographic information; 3D view; checkCIF report

## Figures and Tables

**Table 1:** Hydrogen-bond geometry (Å, °) *Cg*1 and *Cg*2 are the centroids of the N1/C7/C8/C9/C14 and C9–C14 rings, respectively

*D*—H⋯*A*	*D*—H	H⋯*A*	*D*⋯*A*	*D*—H⋯*A*
C17—H17⋯O3^i^	0.93	2.50	3.383 (3)	158
C4—H4⋯*Cg*1^ii^	0.93	2.67	3.635 (4)	127
C4—H4⋯*Cg*2^ii^	0.93	2.76	3.681 (4)	169

Symmetry codes: (i) 
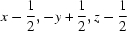
; (ii) 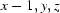.

**Fig. 1.**
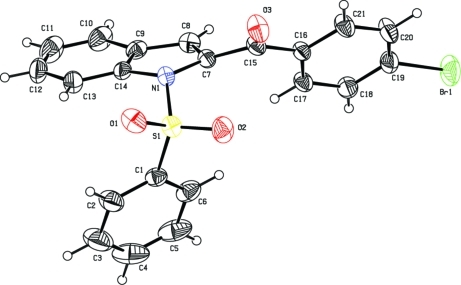
The molecular structure of (I), with atom labels and 30% probability displacement ellipsoids.

**Fig. 2.**
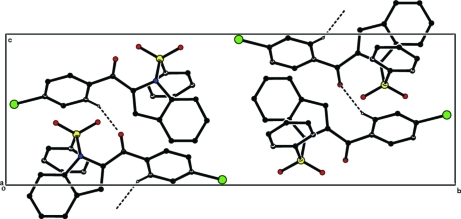
The packing of (I), viewed down the a axis. H-bonds are shown as dashed lines; H atoms not involved in hydrogen bonding have been omitted.

## supplementary crystallographic information

### Comment

Indole derivatives are found abundantly in a variety of natural plants (Poter *et al.*, 1977). Compounds containing the indole moiety exhibit antibacterial and fungicidal activities (Joshi & Chand, 1982). Indole derivatives are also known to exhibit anticancer and anti - HIV (Pomarnacka & Kozlarska-Kedra, 2003) activities.

In continuation of our studies of indole derivatives, we determined the crystal structure of the title compound,(I). The geometric parameters of the molecule of (I) (Fig. 1) agree well with the reported values for similar structures (Chakkaravarthi *et al.*, 2007, 2008). Due to Thorpe–Ignold effect (Bassindale, 1984), bond angles around atom S1 show significant deviation from ideal tetrahedral value, with significant deviations in angles O2—S1—O1 [120.86 (13)°] and N1—S1—C1 [103.79 (11)°].

The phenyl ring forms the dihedral angle of 65.64 (8)° with the indole ring system and the bromophenyl ring makes the dihedral angle of 59.30 (8)° with the indole ring system. The N1—S1—C1 plane is almost orthogonal to both [dihedral angle 72.30 (9)°] indole ring and [dihedral angle 71.68 (12)°] phenyl ring.

The sum of the bond angles around N1 [341.7 (2)°] indicates that N1 atom is *sp*^3^ hybridized (Beddoes *et al.*, 1986). The crystal packing is stabilized by weak intermolecular C—H···O and C—H···π [C4—H4···*Cg*1 (-1 + *x*, *y*, *z*) distance of 3.635 (4)Å and C4—H4···*Cg*2 (-1 + *x*, *y*, *z*) distance of 3.681 (4)Å (*Cg*1 and *Cg*2 are the centroids of the rings defined by the atoms N1/C7/C8/C9/C14 and C9—C14, respectively] interactions.

### Experimental

To a solution of *N*-(2-Formylphenyl)benzenesulfonamide (0.5 g, 1.91 mmol) in dry CH_3_CN (20 ml), K_2_CO_3_ (0.8 g, 5.79 mmol), 2-bromo-1-(4-bromophenyl)ethanone (0.63 g, 2.26 mmol) were added. The reaction mixture was stirred at room temperature for 6 h under N_2_ atmosphere. The solvent was removed and the residue was quenched with ice-water (50 ml), extracted with chloroform (3 × 10 ml) and dried (Na_2_SO_4_). Removal of solvent followed by the residue was dissolved in CH_3_CN (20 ml), Conc. HCl (3 ml) was added. The reaction mixture was then refluxed for 2 h. It was then poured over ice-water (50 ml), extracted with CHCl_3_ (3 × 10 ml) and dried (Na_2_SO_4_). Removal of solvent followed by crystallization from methanol afforded the compound as a colourless crystal.

### Refinement

H atoms were positioned geometrically and refined using riding model, with C—H = 0.93 Å and with *U*_iso_(H) = 1.2*U*_eq_(C).

### Crystal data

**Table:**

C_21_H_14_BrNO_3_S	*F*(000) = 888
*M**_r_* = 440.30	*D*_x_ = 1.542 Mg m^−^^3^
Monoclinic, *P*2_1_/*n*	Mo *K*α radiation, λ = 0.71073 Å
Hall symbol: -P 2yn	Cell parameters from 4249 reflections
*a* = 8.482 (3) Å	θ = 2.5–24.4°
*b* = 25.780 (4) Å	µ = 2.30 mm^−^^1^
*c* = 8.690 (3) Å	*T* = 295 K
β = 93.388 (3)°	Block, colourless
*V* = 1896.9 (10) Å^3^	0.24 × 0.22 × 0.20 mm
*Z* = 4	

### Data collection

**Table:**

Bruker Kappa APEXII diffractometer	4736 independent reflections
Radiation source: fine-focus sealed tube	2944 reflections with *I* > 2σ(*I*)
graphite	*R*_int_ = 0.035
ω and φ scans	θ_max_ = 28.4°, θ_min_ = 1.6°
Absorption correction: multi-scan (*SADABS*; Sheldrick, 1996)	*h* = −9→11
*T*_min_ = 0.609, *T*_max_ = 0.656	*k* = −29→34
18123 measured reflections	*l* = −9→11

### Refinement

**Table:**

Refinement on *F*^2^	Primary atom site location: structure-invariant direct methods
Least-squares matrix: full	Secondary atom site location: difference Fourier map
*R*[*F*^2^ > 2σ(*F*^2^)] = 0.044	Hydrogen site location: inferred from neighbouring sites
*wR*(*F*^2^) = 0.111	H-atom parameters constrained
*S* = 1.03	*w* = 1/[σ^2^(*F*_o_^2^) + (0.0445*P*)^2^ + 0.6759*P*] where *P* = (*F*_o_^2^ + 2*F*_c_^2^)/3
4736 reflections	(Δ/σ)_max_ < 0.001
244 parameters	Δρ_max_ = 0.62 e Å^−^^3^
0 restraints	Δρ_min_ = −0.46 e Å^−^^3^

### Fractional atomic coordinates and isotropic or equivalent isotropic displacement parameters (Å^2^)

**Table:**

	*x*	*y*	*z*	*U*_iso_*/*U*_eq_	
Br1	0.24312 (5)	0.018422 (12)	0.53534 (5)	0.09126 (18)	
S1	0.26404 (8)	0.34303 (3)	0.83609 (7)	0.04855 (18)	
O1	0.3198 (2)	0.38893 (8)	0.9118 (2)	0.0648 (5)	
O2	0.2576 (3)	0.29550 (8)	0.9169 (2)	0.0737 (6)	
O3	0.5440 (2)	0.24395 (7)	0.8349 (2)	0.0671 (6)	
N1	0.3837 (2)	0.33357 (7)	0.6910 (2)	0.0421 (5)	
C1	0.0793 (3)	0.35548 (11)	0.7418 (3)	0.0494 (6)	
C2	0.0204 (4)	0.40492 (12)	0.7428 (4)	0.0703 (9)	
H2	0.0751	0.4312	0.7965	0.084*	
C3	−0.1230 (4)	0.41519 (17)	0.6619 (5)	0.0963 (12)	
H3	−0.1637	0.4487	0.6586	0.116*	
C4	−0.2035 (4)	0.3756 (2)	0.5872 (5)	0.1035 (14)	
H4	−0.3000	0.3824	0.5347	0.124*	
C5	−0.1456 (4)	0.32696 (18)	0.5883 (4)	0.0901 (12)	
H5	−0.2021	0.3006	0.5369	0.108*	
C6	−0.0021 (4)	0.31602 (12)	0.6658 (3)	0.0650 (8)	
H6	0.0386	0.2825	0.6665	0.078*	
C7	0.4047 (3)	0.28357 (9)	0.6256 (3)	0.0410 (5)	
C8	0.4166 (3)	0.28833 (10)	0.4728 (3)	0.0488 (6)	
H8	0.4315	0.2612	0.4045	0.059*	
C9	0.4027 (3)	0.34192 (10)	0.4327 (3)	0.0477 (6)	
C10	0.4108 (4)	0.36829 (14)	0.2933 (4)	0.0706 (9)	
H10	0.4263	0.3505	0.2024	0.085*	
C11	0.3955 (4)	0.42089 (15)	0.2939 (4)	0.0827 (11)	
H11	0.4003	0.4391	0.2019	0.099*	
C12	0.3731 (4)	0.44741 (12)	0.4272 (5)	0.0769 (10)	
H12	0.3629	0.4833	0.4230	0.092*	
C13	0.3650 (3)	0.42278 (11)	0.5686 (4)	0.0613 (7)	
H13	0.3494	0.4410	0.6587	0.074*	
C14	0.3816 (3)	0.36928 (9)	0.5669 (3)	0.0438 (6)	
C15	0.4561 (3)	0.23788 (10)	0.7209 (3)	0.0471 (6)	
C16	0.4037 (3)	0.18560 (9)	0.6675 (3)	0.0430 (6)	
C17	0.2741 (3)	0.17786 (10)	0.5660 (3)	0.0486 (6)	
H17	0.2187	0.2062	0.5244	0.058*	
C18	0.2266 (3)	0.12794 (11)	0.5260 (3)	0.0566 (7)	
H18	0.1389	0.1226	0.4584	0.068*	
C19	0.3100 (3)	0.08671 (10)	0.5870 (3)	0.0566 (7)	
C20	0.4411 (4)	0.09344 (11)	0.6858 (4)	0.0656 (8)	
H20	0.4980	0.0650	0.7246	0.079*	
C21	0.4865 (3)	0.14285 (10)	0.7260 (3)	0.0586 (7)	
H21	0.5744	0.1478	0.7936	0.070*	

### Atomic displacement parameters (Å^2^)

**Table:**

	*U*^11^	*U*^22^	*U*^33^	*U*^12^	*U*^13^	*U*^23^
Br1	0.1039 (3)	0.04279 (18)	0.1244 (4)	−0.01215 (17)	−0.0165 (2)	−0.00695 (18)
S1	0.0576 (4)	0.0482 (4)	0.0396 (3)	0.0031 (3)	0.0004 (3)	−0.0035 (3)
O1	0.0676 (13)	0.0651 (12)	0.0596 (11)	0.0067 (10)	−0.0144 (10)	−0.0246 (10)
O2	0.0985 (17)	0.0670 (13)	0.0573 (12)	0.0100 (11)	0.0197 (11)	0.0186 (10)
O3	0.0788 (14)	0.0531 (11)	0.0650 (12)	0.0036 (10)	−0.0339 (11)	−0.0042 (9)
N1	0.0464 (12)	0.0357 (10)	0.0439 (11)	0.0020 (9)	−0.0010 (9)	0.0010 (8)
C1	0.0446 (15)	0.0566 (15)	0.0475 (14)	−0.0042 (12)	0.0073 (12)	−0.0093 (12)
C2	0.0522 (18)	0.066 (2)	0.092 (2)	0.0062 (15)	−0.0045 (16)	−0.0181 (17)
C3	0.059 (2)	0.103 (3)	0.125 (3)	0.022 (2)	−0.008 (2)	−0.009 (3)
C4	0.045 (2)	0.163 (5)	0.102 (3)	−0.002 (3)	−0.0044 (19)	−0.031 (3)
C5	0.054 (2)	0.129 (4)	0.088 (3)	−0.031 (2)	0.0063 (18)	−0.041 (2)
C6	0.0609 (19)	0.0685 (19)	0.0671 (19)	−0.0180 (15)	0.0171 (16)	−0.0198 (15)
C7	0.0391 (13)	0.0378 (12)	0.0452 (14)	0.0000 (10)	−0.0038 (10)	−0.0029 (10)
C8	0.0482 (15)	0.0539 (15)	0.0439 (15)	−0.0019 (12)	−0.0018 (12)	−0.0090 (12)
C9	0.0382 (14)	0.0604 (16)	0.0438 (14)	−0.0065 (12)	−0.0029 (11)	0.0067 (12)
C10	0.071 (2)	0.086 (2)	0.0539 (18)	−0.0102 (17)	−0.0008 (15)	0.0200 (16)
C11	0.079 (2)	0.095 (3)	0.073 (2)	−0.014 (2)	−0.0080 (18)	0.041 (2)
C12	0.067 (2)	0.0518 (17)	0.110 (3)	−0.0056 (15)	−0.0073 (19)	0.0329 (19)
C13	0.0565 (18)	0.0475 (15)	0.079 (2)	−0.0009 (13)	−0.0005 (15)	0.0073 (14)
C14	0.0349 (13)	0.0428 (13)	0.0533 (15)	−0.0032 (10)	−0.0025 (11)	0.0084 (11)
C15	0.0449 (14)	0.0445 (13)	0.0508 (15)	0.0057 (11)	−0.0058 (12)	−0.0023 (11)
C16	0.0421 (14)	0.0387 (12)	0.0475 (14)	0.0047 (10)	−0.0035 (11)	0.0000 (10)
C17	0.0422 (14)	0.0431 (13)	0.0590 (16)	0.0047 (11)	−0.0100 (12)	−0.0004 (11)
C18	0.0470 (16)	0.0503 (16)	0.0707 (18)	−0.0045 (12)	−0.0114 (14)	−0.0017 (13)
C19	0.0585 (17)	0.0387 (13)	0.0719 (19)	−0.0029 (12)	−0.0005 (14)	−0.0020 (12)
C20	0.071 (2)	0.0425 (15)	0.080 (2)	0.0129 (14)	−0.0178 (16)	0.0013 (14)
C21	0.0556 (17)	0.0489 (15)	0.0685 (18)	0.0082 (13)	−0.0212 (14)	0.0000 (13)

### Geometric parameters (Å, °)

**Table:**

Br1—C19	1.895 (3)	C8—H8	0.9300
S1—O2	1.415 (2)	C9—C14	1.384 (4)
S1—O1	1.4211 (19)	C9—C10	1.394 (4)
S1—N1	1.682 (2)	C10—C11	1.362 (5)
S1—C1	1.754 (3)	C10—H10	0.9300
O3—C15	1.215 (3)	C11—C12	1.368 (5)
N1—C14	1.417 (3)	C11—H11	0.9300
N1—C7	1.424 (3)	C12—C13	1.388 (4)
C1—C2	1.369 (4)	C12—H12	0.9300
C1—C6	1.376 (4)	C13—C14	1.386 (3)
C2—C3	1.394 (5)	C13—H13	0.9300
C2—H2	0.9300	C15—C16	1.485 (3)
C3—C4	1.370 (5)	C16—C17	1.382 (3)
C3—H3	0.9300	C16—C21	1.387 (3)
C4—C5	1.346 (6)	C17—C18	1.387 (4)
C4—H4	0.9300	C17—H17	0.9300
C5—C6	1.385 (5)	C18—C19	1.367 (4)
C5—H5	0.9300	C18—H18	0.9300
C6—H6	0.9300	C19—C20	1.375 (4)
C7—C8	1.343 (3)	C20—C21	1.370 (4)
C7—C15	1.490 (3)	C20—H20	0.9300
C8—C9	1.428 (4)	C21—H21	0.9300
			
O2—S1—O1	120.86 (13)	C11—C10—C9	118.2 (3)
O2—S1—N1	106.80 (11)	C11—C10—H10	120.9
O1—S1—N1	105.58 (12)	C9—C10—H10	120.9
O2—S1—C1	109.31 (14)	C10—C11—C12	121.2 (3)
O1—S1—C1	109.10 (12)	C10—C11—H11	119.4
N1—S1—C1	103.79 (11)	C12—C11—H11	119.4
C14—N1—C7	106.31 (19)	C11—C12—C13	122.5 (3)
C14—N1—S1	119.67 (16)	C11—C12—H12	118.7
C7—N1—S1	121.71 (16)	C13—C12—H12	118.7
C2—C1—C6	121.2 (3)	C14—C13—C12	115.8 (3)
C2—C1—S1	118.9 (2)	C14—C13—H13	122.1
C6—C1—S1	119.9 (2)	C12—C13—H13	122.1
C1—C2—C3	118.8 (3)	C9—C14—C13	122.3 (2)
C1—C2—H2	120.6	C9—C14—N1	108.3 (2)
C3—C2—H2	120.6	C13—C14—N1	129.4 (2)
C4—C3—C2	119.6 (4)	O3—C15—C16	122.0 (2)
C4—C3—H3	120.2	O3—C15—C7	119.8 (2)
C2—C3—H3	120.2	C16—C15—C7	118.1 (2)
C5—C4—C3	121.3 (4)	C17—C16—C21	119.0 (2)
C5—C4—H4	119.4	C17—C16—C15	122.8 (2)
C3—C4—H4	119.4	C21—C16—C15	118.1 (2)
C4—C5—C6	120.2 (3)	C16—C17—C18	120.2 (2)
C4—C5—H5	119.9	C16—C17—H17	119.9
C6—C5—H5	119.9	C18—C17—H17	119.9
C1—C6—C5	119.0 (3)	C19—C18—C17	119.2 (2)
C1—C6—H6	120.5	C19—C18—H18	120.4
C5—C6—H6	120.5	C17—C18—H18	120.4
C8—C7—N1	109.3 (2)	C18—C19—C20	121.7 (3)
C8—C7—C15	125.8 (2)	C18—C19—Br1	119.3 (2)
N1—C7—C15	122.2 (2)	C20—C19—Br1	119.0 (2)
C7—C8—C9	108.6 (2)	C21—C20—C19	118.8 (3)
C7—C8—H8	125.7	C21—C20—H20	120.6
C9—C8—H8	125.7	C19—C20—H20	120.6
C14—C9—C10	120.0 (3)	C20—C21—C16	121.1 (2)
C14—C9—C8	107.5 (2)	C20—C21—H21	119.4
C10—C9—C8	132.5 (3)	C16—C21—H21	119.4
			
O2—S1—N1—C14	163.63 (18)	C10—C11—C12—C13	0.1 (5)
O1—S1—N1—C14	−66.5 (2)	C11—C12—C13—C14	0.2 (5)
C1—S1—N1—C14	48.2 (2)	C10—C9—C14—C13	1.2 (4)
O2—S1—N1—C7	26.6 (2)	C8—C9—C14—C13	179.6 (2)
O1—S1—N1—C7	156.43 (18)	C10—C9—C14—N1	−177.8 (2)
C1—S1—N1—C7	−88.8 (2)	C8—C9—C14—N1	0.6 (3)
O2—S1—C1—C2	138.5 (2)	C12—C13—C14—C9	−0.9 (4)
O1—S1—C1—C2	4.3 (3)	C12—C13—C14—N1	177.9 (3)
N1—S1—C1—C2	−107.9 (2)	C7—N1—C14—C9	−0.8 (3)
O2—S1—C1—C6	−42.9 (3)	S1—N1—C14—C9	−143.64 (18)
O1—S1—C1—C6	−177.1 (2)	C7—N1—C14—C13	−179.7 (2)
N1—S1—C1—C6	70.7 (2)	S1—N1—C14—C13	37.5 (3)
C6—C1—C2—C3	−1.5 (5)	C8—C7—C15—O3	127.2 (3)
S1—C1—C2—C3	177.1 (3)	N1—C7—C15—O3	−32.1 (4)
C1—C2—C3—C4	1.8 (6)	C8—C7—C15—C16	−49.7 (4)
C2—C3—C4—C5	−1.1 (7)	N1—C7—C15—C16	151.0 (2)
C3—C4—C5—C6	0.0 (6)	O3—C15—C16—C17	162.2 (3)
C2—C1—C6—C5	0.5 (4)	C7—C15—C16—C17	−20.9 (4)
S1—C1—C6—C5	−178.1 (2)	O3—C15—C16—C21	−16.0 (4)
C4—C5—C6—C1	0.3 (5)	C7—C15—C16—C21	160.8 (2)
C14—N1—C7—C8	0.7 (3)	C21—C16—C17—C18	1.2 (4)
S1—N1—C7—C8	142.63 (19)	C15—C16—C17—C18	−177.0 (2)
C14—N1—C7—C15	163.0 (2)	C16—C17—C18—C19	−0.5 (4)
S1—N1—C7—C15	−55.1 (3)	C17—C18—C19—C20	−0.8 (5)
N1—C7—C8—C9	−0.4 (3)	C17—C18—C19—Br1	178.9 (2)
C15—C7—C8—C9	−161.9 (2)	C18—C19—C20—C21	1.4 (5)
C7—C8—C9—C14	−0.1 (3)	Br1—C19—C20—C21	−178.3 (2)
C7—C8—C9—C10	178.0 (3)	C19—C20—C21—C16	−0.7 (5)
C14—C9—C10—C11	−0.8 (4)	C17—C16—C21—C20	−0.6 (4)
C8—C9—C10—C11	−178.7 (3)	C15—C16—C21—C20	177.7 (3)
C9—C10—C11—C12	0.2 (5)		

### Hydrogen-bond geometry (Å, °)

**Table:**

*Cg*1 and *Cg*2 are the centroids of the N1/C7/C8/C9/C14 and C9—C14 rings, respectively

**Table:**

*D*—H···*A*	*D*—H	H···*A*	*D*···*A*	*D*—H···*A*
C17—H17···O3^i^	0.93	2.50	3.383 (3)	158
C4—H4···Cg1^ii^	0.93	2.67	3.635 (4)	127
C4—H4···Cg2^ii^	0.93	2.76	3.681 (4)	169

Symmetry codes: (i) *x*−1/2, −*y*+1/2, *z*−1/2; (ii) *x*−1, *y*, *z*.
